# CRISPR–Cas9 gRNA efficiency prediction: an overview of predictive tools and the role of deep learning

**DOI:** 10.1093/nar/gkac192

**Published:** 2022-03-29

**Authors:** Vasileios Konstantakos, Anastasios Nentidis, Anastasia Krithara, Georgios Paliouras

**Affiliations:** Institute of Informatics and Telecommunications, NCSR Demokritos, Patr. Gregoriou E & 27 Neapoleos Str, 15341 Athens, Greece; Institute of Informatics and Telecommunications, NCSR Demokritos, Patr. Gregoriou E & 27 Neapoleos Str, 15341 Athens, Greece; School of Informatics, Aristotle University of Thessaloniki, 54124, Thessaloniki, Greece; Institute of Informatics and Telecommunications, NCSR Demokritos, Patr. Gregoriou E & 27 Neapoleos Str, 15341 Athens, Greece; Institute of Informatics and Telecommunications, NCSR Demokritos, Patr. Gregoriou E & 27 Neapoleos Str, 15341 Athens, Greece

## Abstract

The clustered regularly interspaced short palindromic repeat (CRISPR)/CRISPR-associated protein 9 (Cas9) system has become a successful and promising technology for gene-editing. To facilitate its effective application, various computational tools have been developed. These tools can assist researchers in the guide RNA (gRNA) design process by predicting cleavage efficiency and specificity and excluding undesirable targets. However, while many tools are available, assessment of their application scenarios and performance benchmarks are limited. Moreover, new deep learning tools have been explored lately for gRNA efficiency prediction, but have not been systematically evaluated. Here, we discuss the approaches that pertain to the on-target activity problem, focusing mainly on the features and computational methods they utilize. Furthermore, we evaluate these tools on independent datasets and give some suggestions for their usage. We conclude with some challenges and perspectives about future directions for CRISPR–Cas9 guide design.

## INTRODUCTION

The CRISPR–Cas9 system has revolutionized the field of genome editing and promises the ability to examine genetic interactions at their origin and the opportunity to cure severe inherited diseases. Borrowing from the adaptive mechanisms of bacteria, it identifies a specific site by the complementarity between the guide RNA (gRNA) and the DNA target sequence (1). Compared with previous gene editing technologies, such as zinc finger nucleases (ZFNs) and transcription activator-like effector nucleases (TALENs), which bind to DNA sequences by protein-DNA recognition and require substantial protein engineering, the CRISPR–Cas9 system requires only changing the guide sequence (2). Due to its simplicity, it has been rapidly and widely adopted by the scientific community to target and modify the genomes of a vast array of cells and organisms (3).

However, a major challenge in the effective application of the CRISPR system is to be able to identify target sites that can be cleaved efficiently and for which the candidate gRNAs have little or no cleavage at other genomic locations. Therefore, an ideal gRNA should maximize on-target activity (guide efficiency) while also minimizing potential off-target effects (guide specificity). Balancing these two requirements can be a challenging task and as a result, significant effort in recent years has been focused on developing computational tools to assist in the design of gRNAs. These tools are designed to assist researchers in the selection of the best target sites available. In particular, they help them exclude undesirable targets from their experiments based on predicted low efficiency or specificity, saving resources and time (4).

Following the convention introduced in previous studies (4–7), the current gRNA design tools are grouped into three major types: (i) *Alignment-based* or *Candidate-retrieval*, where the suitable gRNAs are aligned and retrieved from the given genome by locating protospacer adjacent motifs (PAM); (ii) *Hypothesis-driven* or *Rule-based*, where the guide activity is predicted according to empirically derived, handcrafted rules (e.g. GC content) and (iii) *Learning-based*, where the gRNAs are scored by models trained on datasets of CRISPR experiments. Reported results suggest that the latter two types of tools perform better than the alignment-based ones, because they take into account many different features (5). For learning-based tools in particular, these features are combined by models that are generated through machine learning (4,7).

Moreover, a shift has been observed recently in the third category. While the initial learning-based tools relied on conventional machine learning methods, several deep learning-based methods have been explored lately for gRNA activity prediction. For instance, convolutional neural networks (CNNs) are attractive solutions for this task, due to their capability of performing automated feature extraction from sequence data. Therefore, the study and evaluation of these novel tools is timely and interesting.

While some studies have focused on gRNA design tools and their evaluation (5,8–10), there still exist key questions that need to be answered:

- Do learning-based tools perform better than hypothesis-driven ones?
- Are deep learning tools more accurate than previous machine learning tools?
- Is there a single best tool for all experiments and cell types? If not, would it be more accurate to combine the best tools into a meta-tool?
- Are gRNA design rules reproducible across different cell types and organisms?
- How can we improve the gRNA efficiency predictions and derive general gRNA design rules for the CRISPR–Cas9 system?

**Figure 1.**
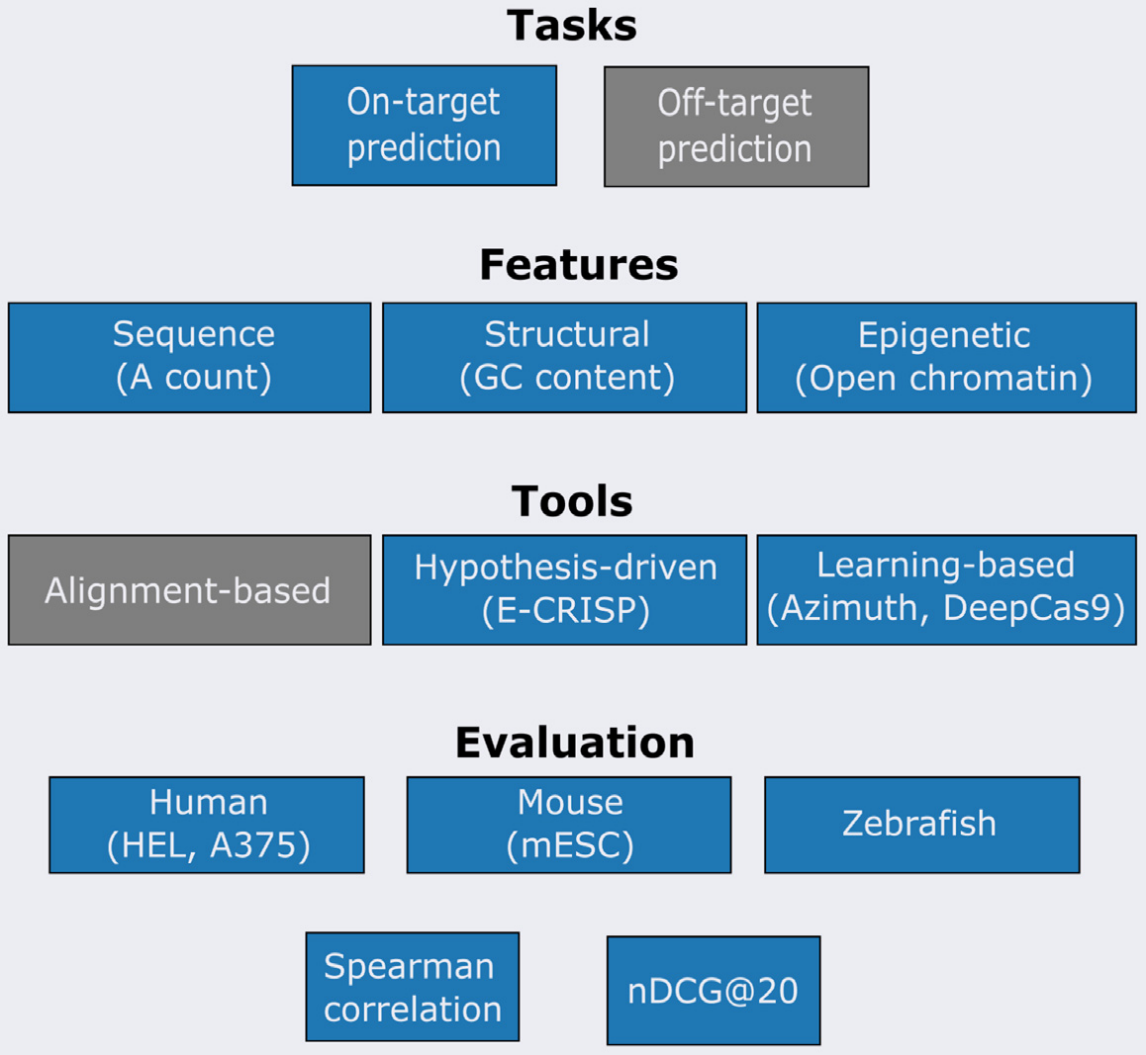
The overall outline of the presented study. We examined the on-target activity problem for CRISPR–Cas9. Features affecting cleavage efficiency are initially described, followed by predictive tools to assist in the guide design process. These tools are then evaluated on six datasets from various organisms (i.e. human, mouse, zebrafish) using two metrics, Spearman correlation and normalized discounted cumulative gain (nDCG). Tasks and tools not examined in the study are colored grey. Examples of features, tools, and datasets are provided in parentheses.

This review addresses these questions, that pertain to the on-target activity problem. Figure 1 presents the overall workflow of our study. First, we present the necessary background for the CRISPR system and we describe the factors that influence gRNA efficiency. Then, we introduce some tools for gRNA activity prediction. Specifically, hypothesis-driven and machine learning models are initially presented, while we also provide a comprehensive overview of the recent deep learning models. Due to space limitations and the rapid development of the genome-editing field, the two former categories are only briefly described. For a more complete overview of those, the reader is referred to existing work (4,9,11). Following their introduction, we evaluate the current gRNA design tools with a focus on the deep learning ones. We conclude with some perspectives about future directions for CRISPR–Cas9 guide design.

## BACKGROUND

Clustered Regularly Interspaced Short Palindromic Repeats (CRISPR) were initially detected as a series of repeated sequences interspaced with short unique sequences in the genome of *Escherichia coli* in 1987 (12). It was later recognized that these short spacer sequences derive from plasmid and viral origins (13). Based on the finding that CRISPR loci are transcribed (14) and the observation that CRISPR-associated (*Cas*) genes encode proteins with putative nuclease and helicase domains (13,15,16), research concluded that the CRISPR-Cas system is an adaptive immune system in archaea and bacteria (17).

**Figure 2.**
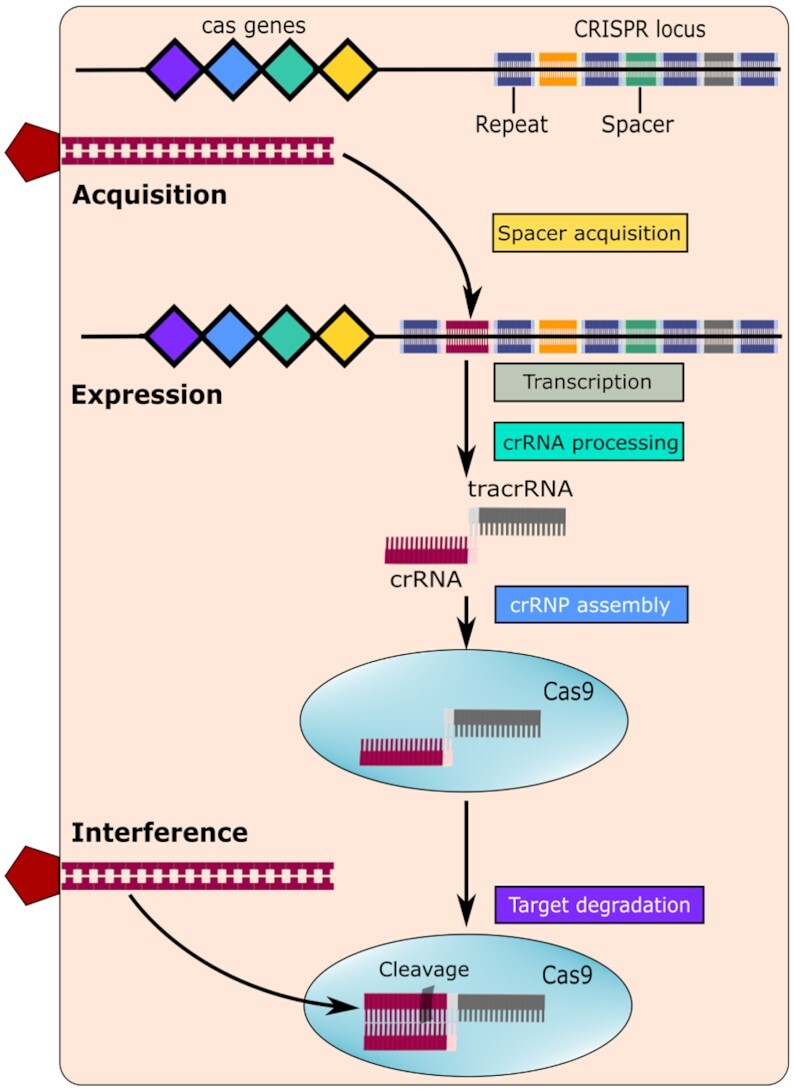
Overview of the CRISPR–Cas9 immune system. Adaptive immunity by CRISPR–Cas systems is mediated by CRISPR RNAs (crRNAs) and Cas proteins, which form multicomponent CRISPR ribonucleoprotein (crRNP) complexes. Three processes underlie the Cas9 system: acquisition, expression, and interference. During acquisition, foreign DNA (red) is incorporated into the CRISPR locus. Expression involves transcribing target DNA into non-coding pre-crRNAs to which trans-activating crRNAs (tracrRNAs) attach, which function as a scaffold for Cas9 binding. During interference, the Cas9 endonuclease uses these sequences to target foreign DNA for cleavage. The components of these processes are indicated based on the involved genes. The non-cas components are presented in grey, while the cas components are colored according to function: spacer acquisition (yellow); crRNA processing (green); crRNP assembly (blue); and target degradation (purple) (18).

Subsequent research has shown that this adaptive immunity occurs in three stages (Figure 2): (i) insertion of DNA sequences from invading viruses or plasmids into the CRISPR locus (known as the acquisition stage); (ii) transcription of the CRISPR array and processing of the precursor transcript into smaller CRISPR RNAs (crRNAs) (known as the expression stage) and (iii) crRNA-directed cleavage of invading DNA by the Cas nucleases (known as the interference stage) (18–20). Within this overall scheme, various CRISPR-Cas systems use different molecular mechanisms to achieve DNA recognition and cleavage. Based on these differences, they are grouped into distinct classes, types and subtypes (21–23). For example, during acquisition, the selection of spacer precursors (protospacers) from the invading DNA appears to be determined by the recognition of protospacer adjacent motifs (PAMs). PAMs are usually a few nucleotides long and differ between variants of the CRISPR-Cas system, such as 5′-NGG-3′ (any nucleotide followed by two guanines) for Cas9 (24,25). Similarly, at the expression stage, the processing of pre-crRNA into mature crRNA is achieved in different ways (26,27). Finally, at the interference stage, the crRNA-guided cleavage can be mediated either by a multi-protein complex or by a single, multi-domain protein. This is the main difference which distinguishes between Class I and Class II CRISPR–Cas systems, respectively (22,28).

**Figure 3.**
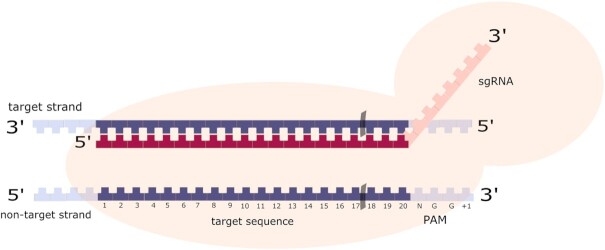
The CRISPR/Cas9 system. Cas9 in complex with sgRNA, containing a gRNA domain (red) and a tracrRNA scaffold (pink), induces a double-stranded break 3 nucleotides upstream of the PAM.

The property of Class II systems to rely on a single protein for cleavage proved to be extremely useful for genome engineering applications (29). In 2012, researchers adapted the *S. pyogenes* Class II CRISPR–Cas9 system (SpCas9) to genome editing and explained its basic mechanism (1). It includes two key components: a synthetic single-guide RNA (sgRNA) and the Cas9 nuclease. The sgRNA is a version of the naturally occurring two-piece guide RNA complex engineered into a single sequence. It consists of the native crRNA that directs Cas9 to the corresponding target site, and a trans-activating crRNA (tracrRNA) which forms a scaffold for Cas9 binding (27). Precise targeting can thus be achieved simply by synthesizing an sgRNA that comprises a guide domain (gRNA) complementary to the target strand and a constant tracrRNA. In the engineered CRISPR–Cas9 system, the gRNA is a 20-nucleotide (nt) sequence at the 5′ end of the sgRNA and is analogous to crRNA in the prokaryotic system (Figure 3) (1).

An important feature of the SpCas9 system is the PAM, which is a CRISPR-dependent and conserved DNA sequence motif adjacent to the target site, and is used by bacteria to distinguish between self and non-self DNA (24). Therefore, target recognition requires both base pairing to the gRNA sequence and the presence of the PAM (i.e. 5′-NGG-3′) adjacent to the targeted sequence (1). When the Cas9 binds with PAM and the target site pairs with the gRNA, a double-strand break (DSB) is caused between positions 17 and 18 of the 20-nt gRNA sequence (Figure 3) (1). Following the break, random insertions or deletions (indels) can be generated via the non-homologous end-joining (NHEJ) pathway, which is error-prone (gene knockout). Alternatively, a desired modification can be introduced through homology-directed repair (HDR) when provided with a DNA template (gene knock-in) (30,31).

## PREDICTING ON-TARGET ACTIVITY

The efficiency of DNA cleavage, both on-target and off-target, depends not only on the intrinsic nuclease activity, but also on target site accessibility and the affinity of DNA binding domain(s) (e.g. gRNA) to the target sequence. However, there is a lack of understanding on the exact behavior of the engineered Cas9 nuclease in living cells, especially regarding the dynamics of its interaction with DNA, and the cell cycle-dependent cleavage activity. Due to the limited biological knowledge, prediction of nuclease target accessibility and cleavage rates in living cells remains difficult. Therefore, experimental validation of target-site selection is necessary. Computational approaches can analyze and extract knowledge from large-scale CRISPR screens. Thus, they can help identify gRNA features modulating Cas9 activity as well as make plausible hypotheses regarding its mechanism of action.

Various potential modulators (features) have been discovered; the following sections discuss the most significant and consistent features affecting cleavage efficiency, a summary of which can be found in Table 1.

**Table 1.** Guide RNA features that could influence on-target activity

Categories	Efficient features	Inefficient features
*Overall nucleotide usage*	A count	U, G count
	A in the middle	GG, GGG count
	AG, CA, AC, UA count	UU, GC count
*Position-specific nucleotides*	G in position 20	C in position 20
	A in position 20	U in positions 17–20
	G, A in position 19	G in position 16
	C in position 18	T in PAM (TGG)
	C in position 16	G in position +1 (NGGG)
	C in PAM (CGG)	
*Motifs*	NGG PAM (esp. CGGH)	poly-N (esp. GGGG)
		TT, GCC at the 3′ end
*Structural*	GC content 40–60%	GC > 80% or GC < 35%
	High GC content in positions 4–8	Stable self-folding
	High GC content in positions 15–20	Stable DNA/RNA duplex
	Low *T*_*m*_ in the middle	
	Accessibility in positions 18–20	
*Epigenetic*	Target with open chromatin	Methylated, inaccessible target
*Miscellaneous*	Target near N-terminus of protein	Target introns, 5′/3′ UTRs
	High Cas9-gRNA concentration	Low Cas9–gRNA concentration

*T*_m_: melting temprature, UTRs: untranslated regions, H: adenine/cytosine/thymine. Adapted from (32).

### Sequence features

In addition to determining the precise cleavage location, the gRNA sequence is one of the most important determinants of cleavage efficiency (33–41).

#### Protospacer adjacent motif (NGG)

The canonical protospacer adjacent motif (PAM) of the CRISPR–Cas9 system is an 5′-NGG-3′ trinucleotide sequence, that is, any nucleotide (N) followed by two guanines (G) (1). Specific interactions between Cas9 and the PAM are required for effective binding of the target sequence and local strand separation prior to cleavage. Even a single mismatch in the PAM results in reduced Cas9 activity, so gRNAs should always be designed to target DNA sequences containing an NGG PAM (39). While the identity of the N is flexible, studies have reported that cytosines (C) are favored and thymines (T) are disfavored in this position (33,36). An extended PAM may also be considered by including the next nucleotide downstream from the PAM sequence. In this additional position, G should be avoided (33,35).

#### gRNA sequence motifs

In most CRISPR applications, a 20-nt DNA oligonucleotide (oligo) representing the guide sequence is cloned into an expression vector and expressed as the gRNA domain within the sgRNA. Thus, the efficiency of both DNA oligo synthesis and the subsequent transcription process affect CRISPR activity. In particular, certain sequence motifs have been identified in the gRNA, which interfere at these stages. Thus, researchers should carefully consider whether the following features apply to the experimental methods used in oligo synthesis and transcription, especially for RNA polymerase-specific effects. For example, a bias against gRNAs initiating with the dinucleotide 5′ AG has been observed, possibly due to 5′ end transcript heterogeneity from in vitro transcription, using T7 polymerase (38,42).

Repetitive bases (i.e. a stretch of identical contiguous bases) could also influence DNA oligo synthesis (36). Specifically, cleavage efficiency is significantly decreased when the gRNA contains a poly-N motif, defined as five contiguous A, five contiguous C, four contiguous G, or four contiguous uracils (U). Furthermore, a GGGG sequence is especially correlated with poor CRISPR activity, not only due to poor oligo synthesis but also because of the propensity to form a special secondary structure (guanine tetrad) which makes the guide sequence less accessible to target sequence recognition. On the other hand, a UUUU motif can also interfere with transcription by RNA polymerase III which terminates upon recognition of a repetitive U sequence (43). Cleavage efficiency is also decreased when the 3′ end of the target sequence has a ‘TT motif’, defined as either a TT-dinucleotide and at least one pyrimidine (TT + Y) or four pyrimidines with at least two Ts (2T + 2Y) (44). This could potentially be related to the tetra-T sequence at the 5′ end of the scaffold sequence. The TT motif could extend the T-rich sequence, resulting in a transcription termination signal and decreased gene editing. At the same position, a ‘GCC motif’ decreases efficiency at the Cas9 targeting stage, irrespective of transcription. The potential underlying mechanisms range from inefficient loading over non-specific binding to off-targets to co-factor-dependent mechanistic problems (44).

#### Overall nucleotide usage

Total nucleotide counts have revealed significant differences between functional and nonfunctional gRNAs, together with some conflicting results. In particular, adenine (A) content has the most significant mononucleotide contribution, with functional gRNAs containing a greater number of As (36). This is largely due to a regional preference in As toward the middle third of the gRNA, around positions 9–16 (33–35). The higher A content in this region corresponds to greater gRNA affinity to Cas9, as shown in Cas9 loading assays (34). On the other hand, U and G content tend to be significantly less represented in functional gRNAs. Contrary to the above, Moreno-Mateos *et al.* (38) discovered based on Zebrafish data, that G enrichment and A depletion contribute to the stability, loading, and activity of gRNA. Excluding the stability factor, the first and last positions of effective gRNAs were found to be strongly G-enriched and C-depleted, while A and U were broadly absent from the gRNA sequence profile. These findings contradict those of previous studies, possibly due to the different organism being studied. To add to the confusion, Rahman *et al.* found that active sgRNAs are G rich as well as A rich (45).

Regarding dinucleotides and trinucleotides, Wong *et al.* (36) observed that both GG and GGG were significantly depleted in functional gRNAs, while Malina *et al.* (46) discovered that too many PAMs within the target sequence inhibit the CRISPR–Cas9 activity in vivo. Consistent with the mononucleotide biases, UU and GC dinucleotides are disfavored in functional gRNAs, while the significantly favored dinucleotides contain an A, namely, AG, CA, AC and UA. However, there is no significant preference for AA, suggesting that the bias for A in the middle of the gRNA requires some degree of nucleotide heterogeneity.

#### Position-specific nucleotide composition

Nucleotide usage at each individual sequence position reveals positions that are critical for efficient editing. Nucleotides proximal to the PAM sequence tend to be the most significant, consistent with the observation that this region is crucial for target interrogation and Cas9 loading (34,35,47). This feature-rich region is known as the *seed region* and corresponds to positions 16–20 of the gRNA (48). For example, U is disfavored at each of these four positions, consistent with the fact that multiple Us in the spacer cause low sgRNA expression (33,36). The most impactful nucleotide seems to be at position 20, where G is strongly preferred and C is strongly disfavored (33,36,37). Adenine is also favored, indicating an overall preference for purines at position 20. Similarly, there is a preference for purines at position 19. On the other hand, C is preferred at position 18, which is the CRISPR–Cas9 cleavage site (35). At position 16, there is a similar trend in which C is enriched and G is disfavored. However, there does not seem to be a clear nucleotide preference for position 17, with some studies showing preference for G while others show C enrichment and G depletion (33,35).

### Structural features

RNA secondary structure is important in many biological processes, since it can determine the nucleotide accessibility and resulting interactions at each locus. Previous studies investigating the role of structural accessibility in RNA-guided target-site recognition have confirmed this hypothesis (49). Likewise, overall secondary structure, self-folding free energy, and the accessibility of individual nucleotides are important features of sgRNA design (36).

**Figure 4.**
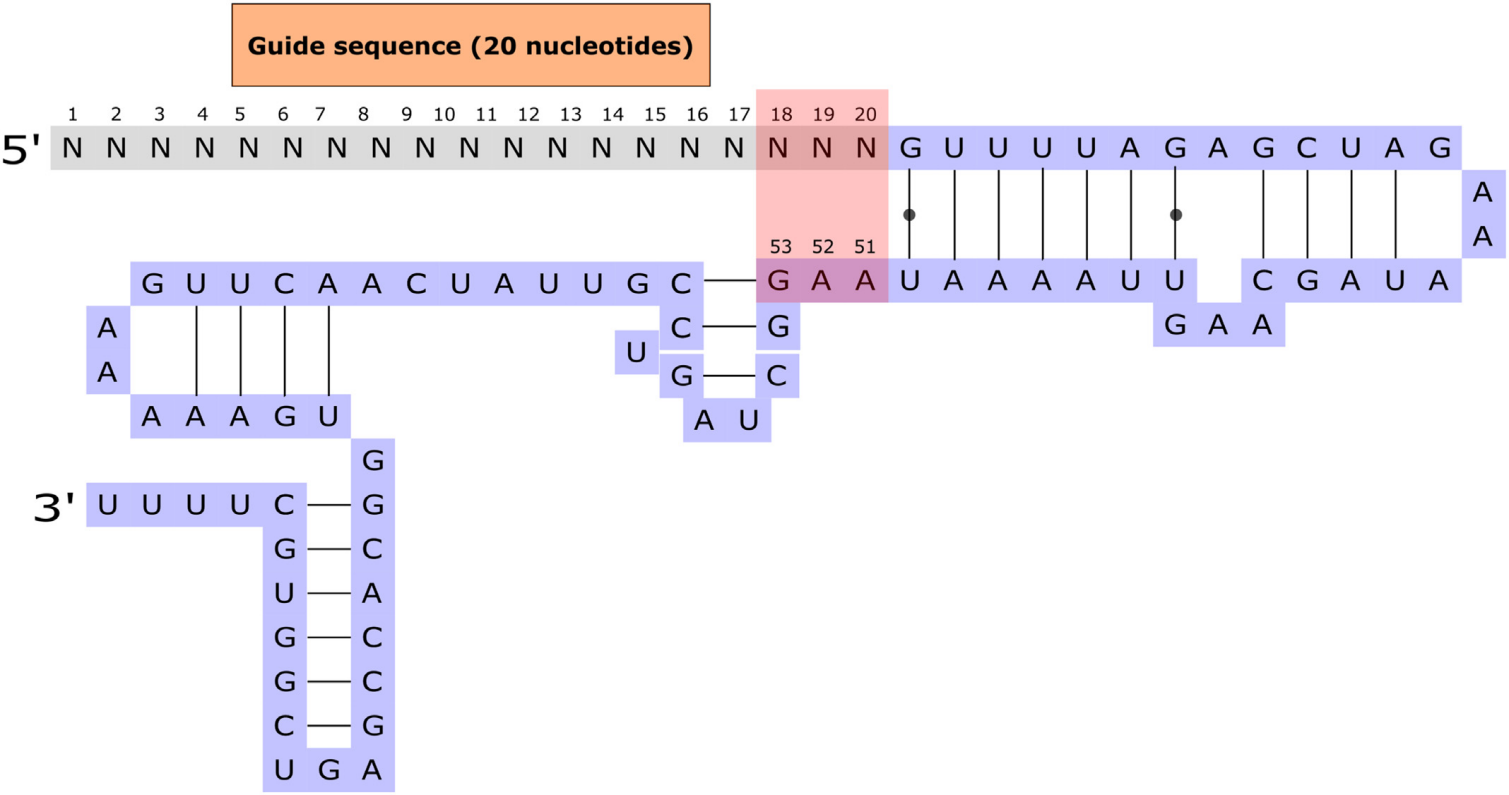
Schematic representation of the sgRNA structure. The guide sequence is complementary to the target sequence and resides at the 5′ end of the sgRNA. The highlighted nucleotides could potentially base pair, leading to an extended stem-loop structure. Black dots indicate weak bonding.

First, the accessibility of certain nucleotides is significantly different between efficient and inefficient sgRNAs. The most impactful difference involves nucleotides at positions 18–20 and positions 51–53 (36). These positions correspond to the 3′ end of the guide sequence and a region of the scaffold RNA, respectively. Interestingly, one conserved motif in the sgRNA consists of a stable stem-loop secondary structure between the nucleotides at positions 21–50 (50). This conserved structure aligns positions 18–20 with 51–53 in an antiparallel configuration (Figure 4). Therefore, base-pairing can potentially occur between bases 19–20 and 52–53 if the sequences are complementary, resulting in an extended stem-loop structure encompassing positions 18–53 (36). This alteration in secondary structure could impede either gRNA seed interactions with the target DNA or scaffold interactions with the Cas9 protein, resulting in a decrease in efficiency. Since the scaffold sequence in positions 52–53 is AA, T nucleotides at the 3′ end of the gRNA could lead to additional base-pairing, resulting in decreased accessibility. This structure-related feature may also explain the observed disfavor of T at the positions closest to the PAM.

Furthermore, the overall structural stability of the guide sequence alone can be evaluated through thermodynamic analysis. Specifically, the self-folding free energy of the gRNA can be used to determine the propensity to form secondary RNA structures through intramolecular interactions. Nonfunctional gRNAs tend to have a more negative self-folding free energy, corresponding to a greater propensity for self-folding, compared to functional gRNAs (36). This suggests that high gRNA stability does not help Cas9 activity. On the other hand, the stability of the DNA/RNA hybrid in the R loop formation can also impact Cas9 activity. Strong DNA/RNA duplex binding is indicative of lower editing efficiency.

The thermodynamic stability of the RNA can also be approximated by the GC content of the sequence, due to the additional hydrogen bond in a GC pair compared to an AT pair. Consistent with the free energy calculation, inefficient sgRNAs have higher GC content on average compared to efficient sgRNAs. However, gRNAs with lower GC content tend to be less active too (33,34), and therefore, some GC content is required. Experiments have shown that GC content in the range of 40–60% seems optimal (33,34,51). Regional GC content could also be important for Cas9 activity due to local interactions of the gRNA. When considering GC content for 10-nt sub-sequences, the strongest predictor of efficiency is high GC content in the first ten bases (40). At a resolution of five nucleotides, higher GC content in positions 4-8 is most indicative of higher activity. In contrast, there is a preference for lower melting temperature in the middle of the gRNA, possibly related to the enrichment in A nucleotides noted in (52). Finally, the six nucleotides proximal to the PAM favor higher GC content which is consistent with the position-specific nucleotide preferences.

The actual overall length of the gRNA has also been shown to influence its activity. Shorter sequences such as 17- or 18-nt have higher specificity but lower efficiency than 19- or 20-nt spacers. On the other hand, gRNAs longer than 20 nucleotides are less effective (35,38).

### Epigenetic features

In addition to the properties of the gRNA and target sequence, the molecular environment in which gene editing occurs could also affect Cas9 activity. For example, the epigenetic features of target sequences are independent of the other gRNA features. These additional features may account for differences in the efficiency of the same gRNA sequence in distinct cell populations.

In particular, local chromatin structure has been identified as a major factor that influences the ability of Cas9 to find the PAM and begin to bind DNA with the seed region of the gRNA (37,48). For instance, Doench *et al.* (33) observed that the N’-terminus of CD15 was a less effective target site, perhaps reflecting gene-specific patterns. However, subsequent work (38) did not confirm a strong effect of chromatin accessibility on CRISPR/Cas9 activity.

Differences between cell types can also be attributed to the assays that have been used to capture the chromatin state, as well as the variable or modest influence of a particular epigenetic feature on each cell. For instance, DNA CpG methylation assays could reflect an aspect of chromatin accessibility not fully captured by DNase I hypersensitivity (DHS). Thus, multiple assays could better explain the binding variation when dealing with similar sequences (48). This issue was studied by Haeussler *et al.* (8) using datasets where the assay was repeated in a different cell type. They observed that the measured efficiencies were highly correlated for some cell type combinations, but not for all. For instance, the Spearman correlation between the knock-out efficiency of 2076 guides in HL60 and KBM-7 cell lines was 0.752. On the other hand, the correlation of the 177k library screens between RPE1 and HCT116 cells was 0.531 and 0.585 for the two replicates. Overall, even if the variation is attributed fully to differences in the chromatin state, its influence is relatively modest, in the range of 10-20% of the rank correlation.

To further illustrate the contribution of epigenetic features, we studied four public datasets that measure the efficiency of gRNAs in human cells. These datasets were included in the study of Chuai *et al.* (41) and were originally provided by Zhang *et al.* (53). They included sequence features, as well as four epigenetic features (i.e. CTCF, H3K4me3, DNase, RRBS), which we used to compare the corresponding cleavage efficiency. Our goal was to confirm the observation of Haeussler *et al.* (8) regarding the contribution of chromatin to the measured efficiency. For this reason, we performed a small-scale experiment, focusing on identical sequences that differed in at least one epigenetic characteristic. The results of this comparison are shown in Table 2. In total, we found 4351 identical gRNAs between all the pairs of the four human cell lines; 1784 of those had at least one different epigenetic feature and a different numerical efficiency (Supplementary Tables S1–S3, Supplementary Note S1). Based on this analysis and the findings of previous studies (8), we conjecture that epigenetic characteristics have a significant, albeit variable, influence on gRNA activity. In order to confirm this, one would need to perform a similar analysis across hundreds of cell lines using CRISPR screen data.

**Table 2.** Correlation of efficiency between identical sequences with different epigenetic features

	Number of	Spearman
Cell line pairs	identical sequences	correlation
Total	1784	0.560
HELA-HL60	69	0.462
HCT116-HL60	40	0.560
HCT116-HELA	1675	0.556
HEK293T-HL60	-	-
HEK293T-HELA	-	-
HEK293T-HCT116	-	-

Cell line pairs that have no identical sequences are left blank.

### Miscellaneous features

#### Target sequence location

When the experimental outcome is measured by the alteration of gene expression or gene knockout, it is important to consider whether cleavage at a specific genomic locus is likely to functionally disrupt the gene. In this respect, relevant studies observed diminished activity of gRNAs targeting close to the C-terminus, since frameshift mutations close to the end of a protein are less likely to disrupt expression. In addition, gRNAs targeting non-coding regions, as well as 5′- or 3′-untranslated regions (UTRs), are ineffective, although target sites that disrupt splicing can be efficacious (33,34,39).

#### Cas9 concentration

Hsu *et al.* (54) reported that high concentrations of the SpCas9–sgRNA complex increase off-site target effects, whereas lower concentrations of Cas9 increase specificity and reduce on-target cleavage activity.

#### Sense/antisense strand

Examining the target strand as a function of activity yielded conflicting results. Doench *et al.* (33) found no statistically significant difference in contrast to a previously observed slight preference for the non-transcribed strand (34).

## TOOLS FOR GUIDE EFFICIENCY PREDICTION

Out of the three categories of guide design tools, we focused on the latter two (i.e. hypothesis-driven and learning-based ones), because they predict the actual efficiency, rather than simply retrieving candidate guide RNAs. In particular, we studied mainly the more recent deep learning models (for CRISPR–Cas9) and chose one representative tool for the hypothesis-driven and machine learning category, due to space limitations.

### Hypothesis-driven tools

Hypothesis-driven tools use empirically derived rules to predict gRNA activity based on the previously described features. For instance, *CHOPCHOP* (55) initially provided two simple metrics; the GC content of the gRNA—ideally between 40% and 80%—and whether the gRNA contains a G at position 20. Later versions incorporated sequence, structural, and chromatin characteristics to give the user a broad selection of metrics to choose from (56,57).

Among those tools, we selected *E-CRISP* (58) because it is simple to use and efficient. It is a web-based tool that requires no programming knowledge or computing infrastructure and allows for fast iteration through gRNA design and parameter selection. In particular, it allows researchers to design guide RNAs to target any DNA sequence—from single exons to entire genomes—for multiple species and uses.

In order to do that, it identifies target sequences ending with a PAM motif 5′-NGG/NAG-3′ and uses them to propose guide RNAs. It also uses a fast indexing approach to locate binding sites and the alignment program *Bowtie 2* to identify off-target effects. It outputs the successful designs, ranked according to target specificity and efficiency. It also assesses the genomic context (e.g. exons, transcripts, CpG islands) of putative designs and provides an option to re-evaluate given gRNAs for efficiency and specificity. Regarding on-target and off-target predictions, it utilises its own ‘SAE (Specificity, Annotation, Efficacy) Score’ to determine the quality of each gRNA, while Rule Set 1 (33) and Spacer Scoring for CRISPR (SSC) (35) are also included in its results.

For all these reasons and recommendations from previous studies (5,41), we chose *E-CRISP* to represent the hypothesis-driven category. We use its ‘Efficacy Score’ (*E*-score) to compare it to the other learning-based tools.

### Machine-learning tools

The resulting efficiency of a gRNA is a complex interplay of factors such as target sequence, cellular environment and experimental conditions. Thus, a simple rule-based system may not be adequate for choosing target sites and designing CRISPR gRNAs.

On the other hand, machine learning models can capture this complex interplay of parameters. In particular, machine learning can produce CRISPR gene-editing models, from a set of samples (experiments), without explicitly specifying the relationship between the features (target properties) and the label (experimental outcome). In the context of CRISPR–Cas9, the experimental outcome can reflect the target site’s ability to generate indels, induce a specific point mutation, or control gene expression. The model trained by machine learning can be stored and used to predict the outcome of new experiments.

In order to train such a model we need to define appropriate labels, select a generalizable feature set, and choose the proper algorithm for our data. Those decisions during model (and experimental) design can lead to important differences, producing a unique predictive model with distinct features. Therefore, it is crucial to present, albeit briefly, the machine learning methods that have been used so far to predict gRNA activity.

Existing machine learning tools can be grouped into three categories:

- *regression models*, such as linear regression (35,38,40), gradient boosting regression tree (GBRT) (39) and random forests (RF) (59),
- *classification models*, such as logistic regression (33), support vector machines (SVM) (36,37,45,60) and RF (59),
- *ensemble methods*, such as stacking (61) and simple model averaging (52).

Besides the algorithm that is used, current tools also differ in the features that they use. Specifically, while they generally use similar genetic data as input (gRNA sequence, PAM, and/or adjacent nucleotides), they vary the window size applied on the target sequence. The windows can range from 23-nt (37) to 40-nt (35), introducing a form of variability. For instance, *Azimuth 2.0* (39), *CRISPRpred* (45) and *TUSCAN* (59) use a 30-nt window, *CRISPRscan* (38) accepts a 35-nt sequence while *sgDesigner* (61) takes a 26-nt gRNA as input.

Furthermore, tools differ in how they represent the target site in the mathematical model, that is, the feature space. The first studies used combinations of position-specific nucleotides and dinucleotides, global nucleotide counts and GC content. More recent studies have begun to include non-sequence information, such as thermodynamic stability of the gRNA and position of the cut site relative to the transcription start site (TSS) (33,36,39). *Azimuth 2.0* (39) includes positional features like ‘exon targeted’ and ‘position of target in gene’. Although Doench *et al.* (39) demonstrated that such features can improve model performance, they can lead to overspecialization and increased heterogeneity. On the other hand, sequence-only models can predict the efficiency of any guide RNA, without requiring species-specific information. There is, therefore, a trade-off between a general purpose model and an organism-specific one. The data used to train each model also originate from a specific organism and cell type under unique experimental conditions. These factors influence the final model, translating into different design rules and predictions.

*Azimuth 2.0* (39) is a state-of-the-art model from the category of machine learning based ones, which is included in our study. It uses a gradient-boosted regression tree (GBRT) model trained on two datasets (39). These datasets include gRNAs targeting human and mouse genes with different detection methods (flow cytometry and resistance assays). The model includes sequence features, thermodynamic features and location of the target within a gene. By retaining the real-valued normalized ranks and adding features not previously used, it has been shown to achieve good predictive performance (39).

### Deep-learning tools

Recent advances in computing technology have enabled a new generation of machine learning methods, deep learning, which has been applied successfully to a number of unsolved problems. Deep learning includes algorithms such as artificial neural networks (ANNs) that can learn data representations at multiple levels of abstraction (62).

ANNs, initially inspired by neural networks in the brain (63,64), consist of multiple layers of interconnected compute units (neurons). Given this structure, an ANN takes the raw data at the lowest (input) layer and transforms them into increasingly abstract feature representations by successively combining outputs from the preceding layer in a nonlinear way, describing highly complicated functions in the process.

Therefore, deep learning provides an effective approach for learning complex patterns at multiple layers (62). Compared to traditional machine learning methods, deep learning algorithms can extract features from large, annotated datasets, such as images or genomes, and use them to create predictive tools based on patterns hidden in the data (65). Using these algorithms, researchers can bypass the process of feature creation, which is labour-intensive and requires considerable domain knowledge (62).

For this reason, deep learning algorithms have been recently used to manage increasing amounts and dimensions of data generated by high-throughput analyses. For example, ANNs and convolutional neural networks (CNNs) have been successfully applied to predict splicing activity (66), sequence specificities of DNA- and RNA-binding proteins (67), and to study the effect of DNA sequence alterations (68). For more information about the structure and function of CNNs, the reader is referred to Appendix A.

**Figure 5.**
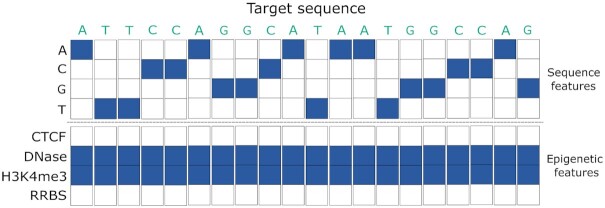
Representation of the target sequence and its corresponding epigenetic features. The latter are only captured by *DeepCRISPR*, *CNN-SVR* and *C-RNNCrispr*.

Being able to uncover underlying patterns in unprocessed data, rather than requiring perfectly curated feature sets, is a useful capability in the CRISPR space. Thus, various deep learning approaches have recently been used for CRISPR gRNA design, including *DeepCRISPR* (41), *DeepCas9* (69), *CRISPRLearner* (70), *DeepSpCas9* (71), *DeepHF* (72), *CNN-SVR* (53) and *C-RNNCrispr* (73). Most of these deep learning models represent the target sequence using One-Hot-Encoding, while some capture the epigenetic features too (Figure 5). The rest of this section presents each CRISPR–Cas9 deep learning model, discussing their commonalities and differences.

*DeepCRISPR* (41) is a novel deep neural network for gRNA on-target knockout efficiency prediction, comprising two parts. The first part is a deep convolutionary denoising neural network (DCDNN)-based autoencoder that learns the underlying representation of gRNA regions in an unsupervised manner. The DCDNN-based autoencoder is followed by a full CNN model for predicting gRNA efficiency. DeepCRISPR adopts a fine-tuning strategy to train the model, utilizing the autoencoder to be part of the classifier and then tunes the whole network (i.e. autoencoder and classifier) with labeled data. The system is trained with both sequence and epigenetic features from four human cell lines (HCT116, HEK293T, HELA, and HL60) containing ∼15 000 gRNAs. In addition, *DeepCRISPR* uses data augmentation to generate novel gRNAs with biologically meaningful labels. This is done by exploiting the observation that mismatches in the first two positions from the 5′ end usually has no effect on cleavage efficacy. The augmentation creates roughly 0.2 million non-redundant gRNAs for the training process, further improving the tool’s performance. The final model can be used for either regression or classification, by using the actual numerical efficiency or a converted binary value, respectively. In summary, *DeepCRISPR* was the first deep learning model for gRNA design that used epigenetic information, augmentation, and representation learning to outperform the state-of-the-art tools across a variety of human datasets.

*DeepCas9* (69) is another deep learning framework based on CNNs that automatically learns the sequence determinants and predicts the efficiency of gRNAs. It accepts a 30-nt sequence, without additional epigenetic features. The sequence is translated into a one-hot-encoded matrix that is used as input by the CNN, essentially skipping the steps of feature extraction and feature selection. The tool also implements an ensemble strategy that combines three deep learning models trained on different datasets (HL60, HEK293T, mEL4) and outputs their weighted average. This approach improves the predictive performance of the final model compared to the individual ones. Thus, the efficiency score of a new sequence is calculated with the following weighted sum:

$$\begin{eqnarray*} DeepCas9_{score} &=& 0.2 * DeepCas9_{mEL4}\\ && + 0.3 * DeepCas9_{293T} \\ &&+ 0.5 * DeepCas9_{HL60}, \end{eqnarray*}$$

where *DeepCas*9_*mEL*4_, *DeepCas*9_293*T*_ and *DeepCas*9_*HL*60_ are the scores predicted by each of the three models, and *DeepCas*9_*score*_ is the final prediction.

*DeepSpCas9* (71) is a regression model that predicts gRNA on-target activities. Like other design tools, it accepts 30-nt sequences and uses a simple CNN architecture to automatically learn their important features and predict their efficiency. However, unlike other tools, it is trained on a large, unique dataset of Cas9-induced indel frequencies. This dataset was created by evaluating the gRNA activities of 12 832 target sequences using a high-throughput approach, based on a human cell library containing single-guide RNA-encoding and target sequence pairs. By training on this dataset, *DeepSpCas9* showed a stable and high performance when tested against independently generated datasets. Therefore, it can be used in different species, organisms, and cell types.

*DeepHF* (72) uses a recurrent neural network (RNN) to predict gRNA activity. In particular, *DeepHF* uses a Bidirectional long short-term memory neural network (BiLSTM) to extract features and combines them with hand-crafted biological features to construct the final model. It also utilizes a fine-tuning strategy to improve the prediction accuracy of the model under different expression conditions (i.e. under a U6 or T7 promoter). Using this approach and training on a dataset of ∼50 000 gRNAs, *DeepHF* has been shown to outperform the current state-of-the-art tools across various datasets.

*CRISPRLearner* (70) borrows ideas from *DeepCRISPR* and *DeepCas9* to create a system with improved performance. In particular, it accepts 23-nt sequences and augments them by changing each one into a new gRNA with two mismatches in the PAM distal region. It then uses those sequences to train 10 models on 10 different datasets. In contrast to *DeepCas9*, *CRISPRLearner* does not output their weighted average but maintains the individual scores of the ten models. The user can then calculate the efficiency of a sequence using one of the 10 models, depending on the organism or cell line of choice. Users can also train their own model using a different dataset, expanding the system and allowing it to predict gRNA efficiencies regarding new cell types and organisms.

*CNN-SVR* (53) combines two major components: a CNN for extracting gRNA sequence/epigenetic features and a Support Vector Regression (SVR) algorithm for predicting gRNA cleavage efficiency. *CNN-SVR* is pre-trained on the same augmented dataset as *DeepCRISPR* and can be fine-tuned for predictions on small cell-line-specific datasets. In addition, it adopts a two-step feature-selection strategy to identify important subsets of the initial CNN features. To be specific, it ranks the importance of features, based on information gain, and then uses sequential forward search (SFS) to determine the optimal set. The selected features are fed into the SVR classifier to complete the gRNA activity prediction.

*C-RNNCrispr* (73) is a hybrid CNN and bidirectional gate recurrent unit (BGRU) network that predicts CRISPR–Cas9 gRNA on-target activity. It uses CNNs to automatically learn the gRNA sequence features and four epigenetic features. BGRU is then used to model the sequential dependencies of the gRNA features. *C-RNNCrispr* is also pre-trained on the same benchmark dataset as *CNN-SVR* and can be fine-tuned on small cell-line datasets to improve predictive performance.

## EVALUATION OF ACTIVITY PREDICTION TOOLS

### Datasets

Several tools are available for CRISPR gRNA design, but only a few of them have been compared on the same task (5,8–10). In particular, previous studies have shown that current models perform reasonably well when trained and tested on different parts of the same data, using cross-validation. However, predictions on independent datasets are less accurate than expected (8). This observation indicates the need to evaluate the tools on independent datasets that differ from the training ones. The independent testing sets could also be used to compare new deep learning tools against the existing ones.

A total of six testing datasets were gathered from published studies, namely: Labuhn (40,41), Shalem (33), Koike-Yusa (8), Xi Xiang (74), Shkumatava (8) and Gagnon (8). To avoid training bias, we only considered independent datasets that were not used to train respective models. The datasets we used come from various cell lines and organisms (human, mouse, zebrafish). They also represent different experimental attributes that need to be considered in the subsequent analysis. Table 3 presents an overview of the testing datasets and their characteristics. The ‘Materials and Methods’ section provides further details.

**Table 3.** Summary of testing datasets

Dataset name	Number of instances	Species/Cell line	Guide RNA promoter	Delivery method	Analysis method
*Labuhn*	424	Human/HEL	U6	Lentivirus	Flow cytometry
*Shalem*	1278	Human/A375	U6	Lentivirus	Resistance assay
*Koike-Yusa*	1064	Mouse/mESC	U6	Lentivirus	Resistance assay
*Xi Xiang*	10 592	Human/HEK293T	U6	Lentivirus	Amplicon sequencing
*Shkumatava*	162	Zebrafish	T7	RNA injection	Sanger sequencing
*Gagnon*	111	Zebrafish	T7	RNA injection	Amplicon sequencing

### Evaluation metrics

Another important aspect of the evaluation process is the choice of metrics. We selected two different metrics to evaluate the current models; namely Spearman correlation and normalized discounted cumulative gain (nDCG), capturing different aspects of the models’ performance.

Spearman correlation evaluates the ability of the models to predict the actual efficiency of each gRNA sequence by comparing the predicted value to the one measured experimentally. Spearman correlation has been used in most of the existing literature (8,39,41,69–71), because the available datasets, as well as the predictions of different models are on substantially different scales. Therefore, while some models are trained to minimize the mean squared error (MSE), the comparison between models and datasets is necessarily done in terms of ranking, using Spearman correlation. Furthermore, since most of the available datasets do not include labels (i.e. efficient versus non-efficient), classification metrics were not directly applicable.

We also used another rank-based evaluation measure, namely nDCG, in order to focus on the retrieval of the most efficient gRNAs and ignore the less efficient ones. In other words, using nDCG, we avoid judging how well a tool is doing in predicting inefficiency.

Thus, our analysis evaluates both the general performance of each model and their ability to identify the most effective gRNAs. Details about the implementation of the evaluation metrics are provided in the ‘Materials and Methods’ section.

### Baselines and bounds

To highlight the differences between the tools being evaluated, we introduce two baseline approaches. The first baseline uses six extreme gradient boost (XGB) models, trained on six different datasets. The final prediction is the average of their predictions, which we denote as *OHE Average*. The second baseline, named simply *Average*, is the mean of *DeepCRISPR*, *DeepCas9*, *DeepSpCas9* and *Azimuth 2.0* predictions.

In addition to the baseline systems, we introduce an upper and lower bound to the correlation coefficients in order to facilitate the interpretation of our results. The upper bound is based on a previous study (8), which showed that the measured efficiencies are not perfectly correlated even for data from the same assay and cell line. Specifically, they showed that for datasets where replicates are available, the Spearman correlation is in the range of 0.71–0.77. This illustrates the quality of the data and suggests that a correlation of ∼0.7 constitutes an upper limit of any prediction.

We also define a lower bound based on the results obtained by the different tools using cross-validation on the training data. In order to determine the bounds, we present the evaluation of each tool in their original study (Table 4). According to these results, we choose a correlation of 0.4 to judge the success of a system in an experiment. If a tool performs better than this on independent datasets, we argue that it has obtained an acceptable prediction ability, and can be used on independent cell lines. We did not take into consideration the results of *CNN-SVR* and *C-RNNCrispr* as they were pre-trained on a benchmark dataset that included all cell lines. Thus, their results are likely biased. The choice of 0.4 correlation is further supported by Haeussler *et al.* (8), who show that such values are high enough to reduce the number of guides in practice, even for genome editing projects of just a few loci.

**Table 4.** Average Spearman correlation coefficients as mentioned in the study that introduced each method

Models	Same cell line	New cell line
*DeepCRISPR*	0.601	0.406 (LOCO)
*DeepCas9*	-	0.351
*DeepSpCas9*	-	0.464
*DeepHF*	0.867	0.433
*CRISPRLearner*	0.432	-
CNN-SVR*	0.714	0.714 (LOCO)
C-RNNCrispr*	0.699	0.692 (LOCO)
*Azimuth 2.0*	0.514	0.462

‘Same cell line’: includes data from the cell line that was used to train the specific model.

‘New cell line’: includes data from new cell line(s).

‘LOCO’: Leave-One-Cell-Out procedure.

*: model has been pre-trained on data including all cell lines.

There are no available data for *E-CRISP*, which is omitted.

### Comparative analysis

**Figure 6.**
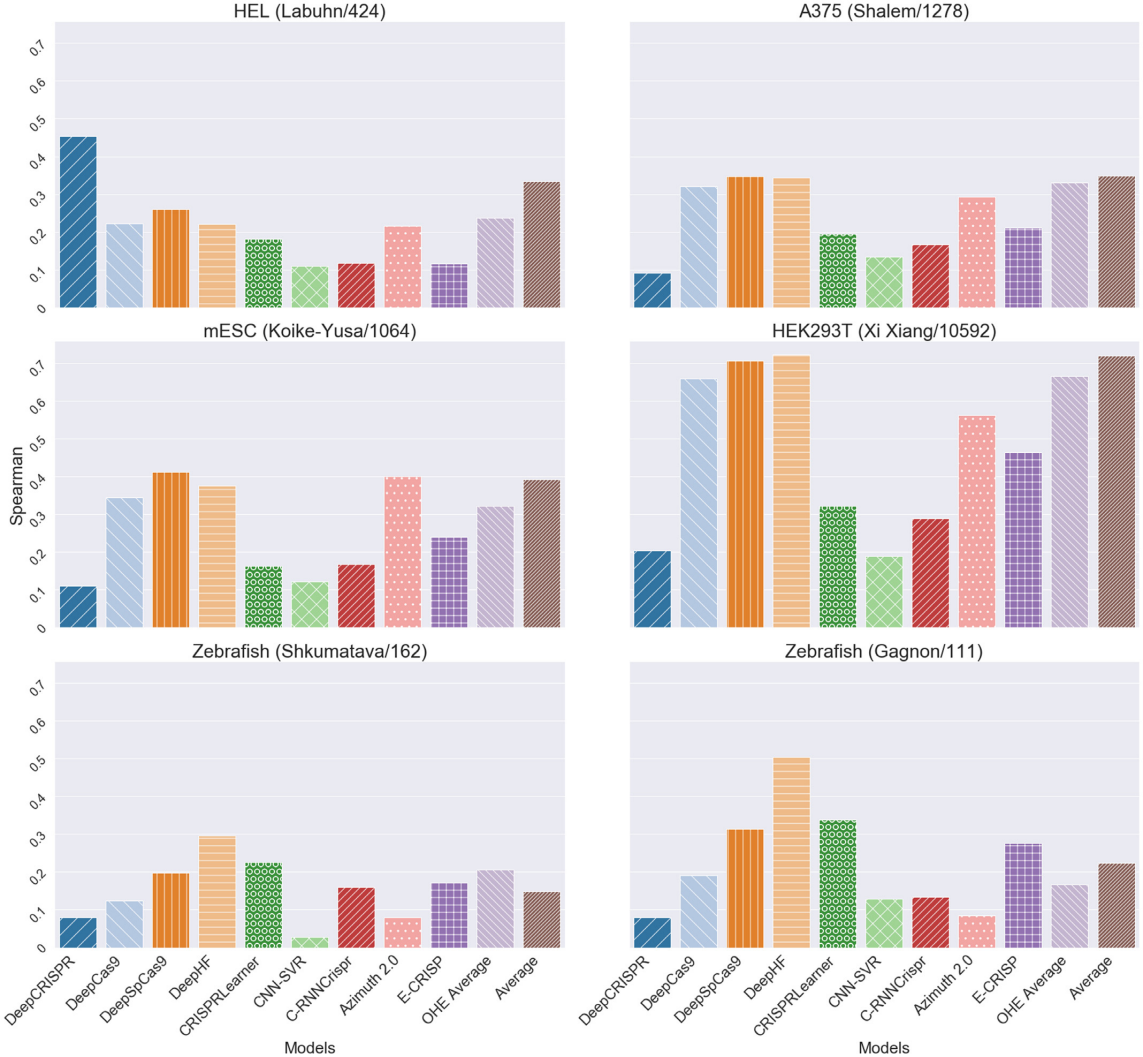
Comparison of gRNA efficiency predictions using Spearman correlation. The name and number of instances for each dataset are shown in parentheses.

In this section, we present the results of our comparison of the eleven different prediction tools. First, we observe that the correlation between predicted and actual efficiency varies considerably (Figure 6). This is especially evident in the case of *DeepCRISPR* which has the best performance in the HEL cell line but performs poorly in the other datasets. Across all datasets, *DeepHF* and *DeepSpCas9* are consistently among the most accurate models, confirming their good general performance. However, the same holds for the baseline models, especially the *Average* one, which is close to or better than *DeepSpCas9* and *DeepHF* in the four cell lines using a U6 promoter (i.e. HEL, A375, mESC, HEK293T).

On the other hand, there are some tools that achieve similar performance across all datasets. These include *CRISPRLearner*, *CNN-SVR* and *C-RNNCrispr*. *CRISPRLearner* performs better than the other two. Specifically for *CRISPRLearner*, it should be noted that every prediction was done by first choosing the most suitable model. This explains why it outperforms the other tools (except *DeepHF*) on zebrafish data, since the selected model was trained on a zebrafish dataset using the same promoter. *DeepHF* was also fine-tuned on a relevant dataset and achieved similarly good performance. Despite individual differences, all design tools performed worse on the datasets that differed from the ones they were trained on (Table 4).

**Figure 7.**
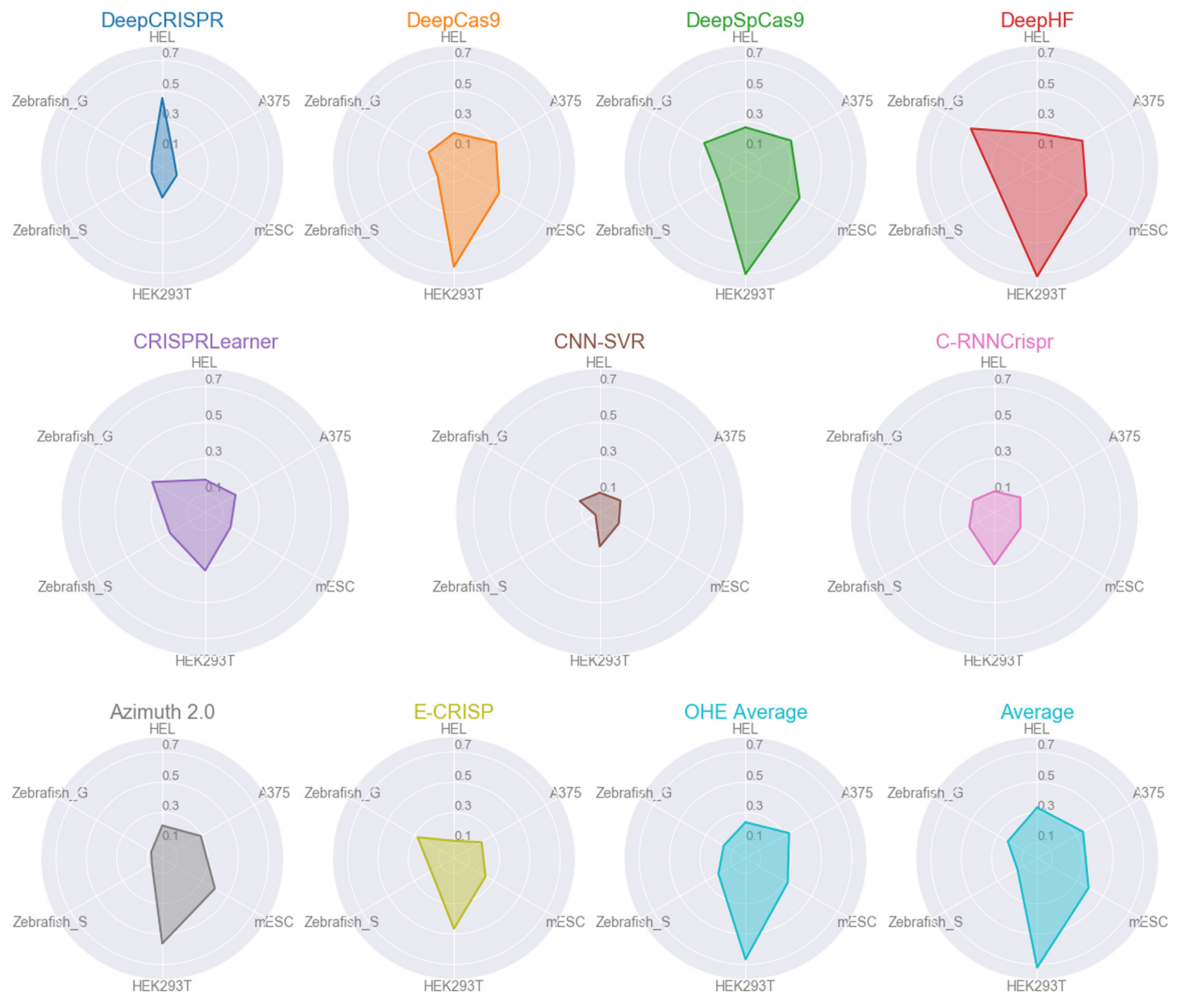
Spearman correlation for each tool and dataset. Each polygon represents a tool and the edges illustrate the obtained correlation for the respective dataset. In general, the larger the polygon area, the better the overall performance of the tool.

Figure 7 provides a different view of the predictive ability of each model. This view illustrates the good and robust performance of *DeepHF*, *DeepSpCas9* and the baseline models more clearly. *DeepCas9*, which is also an average, and *Azimuth 2.0* follow the other three tools in performance. Therefore, conventional machine learning models, such as XGB (*OHE Average*) and GBRT (*Azimuth 2.0*) seem to achieve at least comparable performance to the deep learning ones. Finally, even though *E-CRISP* is more accurate than some learning-based tools (e.g. *CNN-SVR*), it does not achieve high enough correlations. However, it demonstrates a stable performance across all datasets.

**Figure 8.**
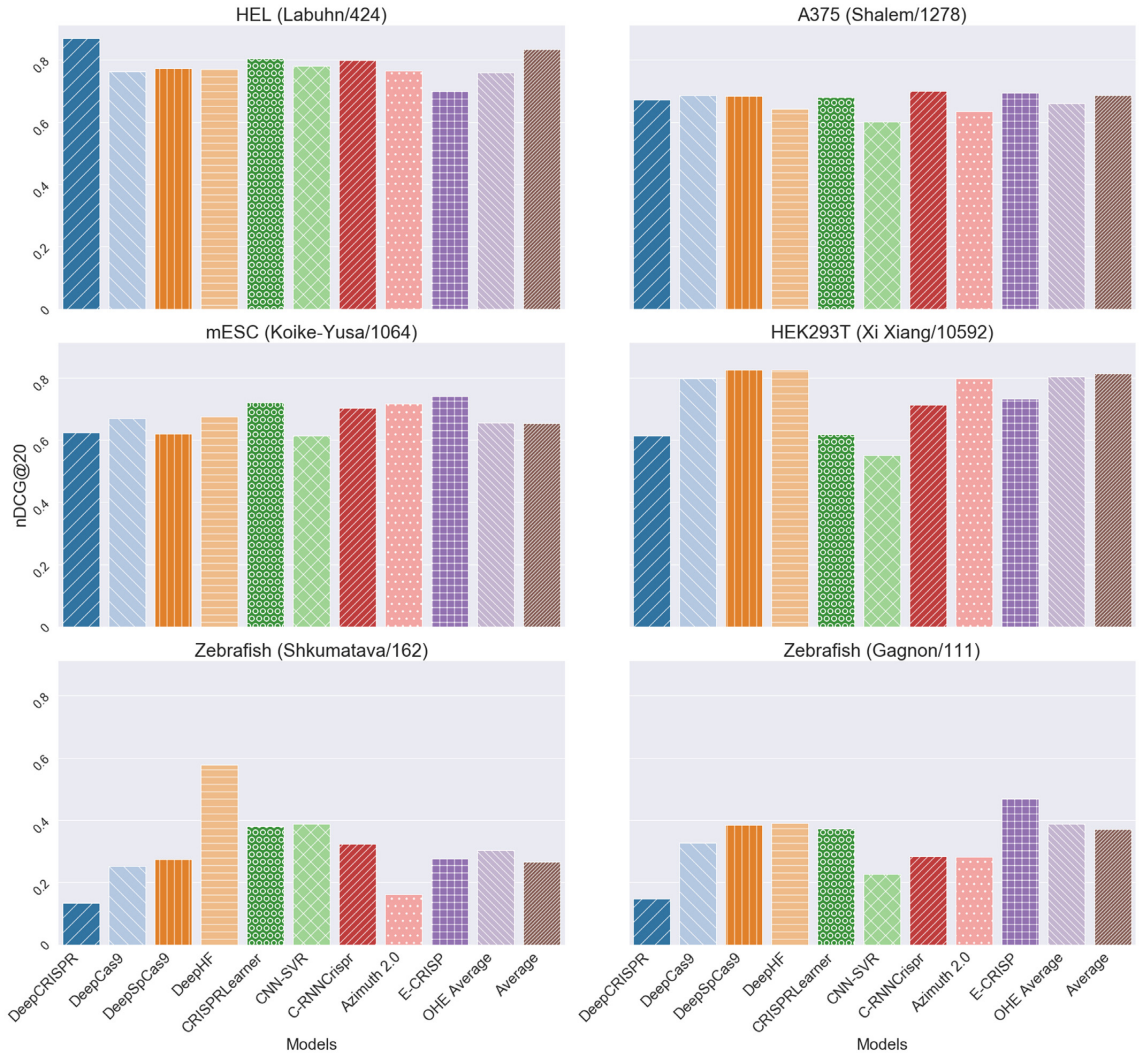
Comparison of gRNA efficiency predictions with nDCG@20. The name and number of instances for each dataset are shown in parentheses.

Performance on the best 20 predictions, according to nDCG@20, is quite different from Spearman correlation (Figure 8). While *DeepCRISPR* still outperforms the other tools on the Labuhn dataset, *E-CRISP* and *CRISPRLearner* achieve better results using this metric. *C-RNNCrispr* also improves, especially on the Labuhn, Shalem, and Koike-Yusa dataset. On the other hand, *DeepHF* and *DeepSpCas9* show a consistently good performance according to both metrics. Therefore, nDCG@20 seems to capture a unique aspect of the models’ ranking ability, which is distinct from the correlation analysis. However, according to nDCG@20, the differences between the models become less evident and the comparison more difficult. For this reason, the radar charts are not helpful in the case of nDCG@20 and we do not illustrate them here. They can be found in the provided Supplementary material (Supplementary Figure S1).

In summary, we conclude that there is no single best model across datasets and metrics. One model may outperform the others according to correlation, but perform poorly when evaluated according to the top ranking ability. Therefore, it is crucial to choose the appropriate metric when comparing different models.

### Case study for gRNA design

In this section, we examine how researchers can use the existing tools and the knowledge about on-target activity in their experiments. In other words, we want to assess the impact of our analysis and how its results can be used to better inform the guide design process. To accomplish this, we present two case studies that involve the treatment of a genetic disorder. In particular, we apply the evaluated tools to select the appropriate gRNAs for each case and illustrate how they can better inform the experimental design. Similar to Peng *et al.* (52), we study the guide design process using retinitis pigmentosa (RP) and X-linked chronic granulomatous disease (X-CGD) as examples. Experiments on these two diseases have been successfully undertaken by domain experts recently (75,76).

Yu *et al.* (75) attempted to knockdown the *Nrl* gene to prevent retinal degeneration in a mouse model and suggested an adeno-associated virus (AAV)-based CRISPR–Cas9 system for gene disruption as a promising treatment option for patients with RP. To accomplish that, they designed five candidate gRNAs (denoted NT1 to NT5) against the mouse *Nrl* coding region. Among those, they selected NT2 for the *in vivo* study based on its higher ability to generate indels and lower predicted off-target potential.

For our comparison, we used *CHOPCHOP* (55) to retrieve all the candidate sequences for *Nrl* gene knockdown in the mouse genome (mm39). In total, 147 potential spacer sequences were found with the PAM 5′-NGG-3′. The cleavage efficiencies of the 147 candidate gRNAs were predicted by the most representative tools. *DeepSpCas9*, *DeepHF*, *Average*, and *DeepCRISPR* were used for their performance based on Spearman correlation, while *CRISPRLearner*, *C-RNNCrispr* and *E-CRISP* were selected according to nDCG@20. Finally, we ranked the predictions of each tool in order to compare the position of the selected gRNA (i.e., NT2). The results of this evaluation are shown in Table 5.

**Table 5.** Rank of the selected guide (NT2) based on each tool’s predictions

Model	Rank	Evaluation performance*
*DeepCRISPR*	87	Variable (SC, nDCG)
*DeepSpCas9*	2	Best (SC, nDCG)
*DeepHF*	2	Best (SC, nDCG)
*Average*	2	Best (SC, nDCG)
*CRISPRLearner*	33	Good (nDCG)
*C-RNNCrispr*	17	Good (nDCG)
*E-CRISP*	2	Stable (SC, nDCG)

* Based on the results of the comparative analysis (Figures 6–8). The corresponding metric is shown in parentheses. SC: spearman correlation, nDCG: normalized discounted cumulative gain.

We observe that the three most accurate tools in our evaluation, *DeepSpCas9*, *DeepHF*, and *Average*, rank the selected gRNA (NT2) very highly (second position). Interestingly, *E-CRISP*, a hypothesis-driven tool with a stable performance, also identifies the efficient guide sequence. However, *DeepCRISPR*, which was the most accurate tool in the HEL cell line but performed poorly in the others, ranked the selected gRNA at position 87. Similarly, *CRISPRLearner* and *C-RNNCrispr*, which demonstrated a good performance based on the nDCG metric but not according to Spearman correlation, did not include the chosen gRNA in their top-ranked guides.

Regarding the second case study, De Ravin *et al.* (76) used CRISPR–Cas9 to repair a mutation in the CYBB gene of CD34+ hematopoietic stem and progenitor cells (HSPCs) from patients with the immunodeficiency disorder X-CGD. Unlike the previous example, the cutting site should be close to the mutation site in order to promote HDR and correct the point mutation. Four guide sequences (gRNA1, gRNA2, gRNA3 and gRNA8) around the CYBB mutation site were evaluated, of which gRNA2 displayed maximal cutting efficiency. Similar to the previous case, we used the evaluated tools to predict the cleavage efficiency of each gRNA. The resulting predictions can be seen in Table 6.

**Table 6.** Predicted efficiency of each gRNA from the De Ravin study

	gRNA2	gRNA3	gRNA8	gRNA1
Actual cleavage	**0.21**	0.0125	0.01	0.004
*DeepCRISPR*	0.099	0.431	0.096	**0.464**
*DeepSpCas9*	**0.629**	0.482	0.122	0.063
*DeepHF*	**0.628**	0.421	0.312	0.363
*Average*	**0.594**	0.437	0.190	0.220
*CRISPRLearner*	**0.559**	0.393	0.255	0.371
*C-RNNCrispr*	**0.202**	0.164	0.189	0.157
*E-CRISP*	**0.530**	0.353	0.270	0.281

The evaluated gRNAs are ordered from most to least efficient. The maximum actual and predicted efficiencies are marked in bold.

We observe that all tools except *DeepCRISPR* identify the most efficient guide sequence. In addition, *DeepSpCas9* ranks all the gRNAs correctly in contrast to the other tools, which swap at least a pair of guides (e.g. gRNA8 and gRNA1 for *E-CRISP*). These findings confirm that tools with an adequate performance in our evaluation can at least identify some of the most efficient gRNAs. On the other hand, tools that demonstrated a biased performance (e.g. *DeepCRISPR*) may miss efficient sequences and even lead to conflicting results. Finally, tools that perform well when evaluated with both metrics (e.g., *DeepSpCas9*) seem to identify correctly both the efficient and the inefficient gRNAs.

The presented use cases suggest that our evaluation can capture the tools’ abilities to identify efficient gRNAs and guide gene editing experiments for various applications. We also illustrate that the top-performing tools can be used to suggest the top-ranked gRNAs (e.g. top 3) and narrow down the scope of the search for efficient guides (i.e. from 147 to 3–5 sequences in our case). Such a recommendation approach can save time and cost, without sacrificing accuracy and efficiency.

## DISCUSSION

### Challenges

Our results show that there is room for improvement in the tools that predict gRNA efficiency. They also highlight several open challenges, that extend to the design process, including both data acquisition and modeling. In this section, we highlight the main challenges and suggest ideas and directions for improvement.

#### Data sparsity and heterogeneity

Sparsity and quality of labeled data are important challenges in the process of training gRNA efficiency predictors. There are relatively few gRNAs with known efficiency as it is expensive to collect such data. Insufficient labeled data make the predictive models inefficient, even when data augmentation techniques are used, as we demonstrate in the evaluation of *DeepCRISPR*.

Given the data sparsity, most of the existing methods have used a single data set or a small number of gRNAs to learn a predictive model. Therefore, their prediction and guide design rules are likely to be biased or incomplete. For example, in the study of Xu *et al.* (35) the sequence determinants selected for HL60 and mESC cells were not identical. In the same study, the authors recognized that some cell-specific sequence preferences may be missed. To overcome the bias of a single data set and predict gRNA efficiency more accurately, some recent tools combine several of the available datasets. However, the different ways of combining the datasets can lead to different results.

Even when experimentally labeled data are available, their use for supervised learning should be done with great care. In CRISPR gene-editing experiments, the outcome can be measured with various methods. Often, the label is often gene knockout efficiency, such as the amount of gene expression measured by fluorescence. In particular, green fluorescent protein (GFP)-based and drug-resistance assays can capture functional gene knockout, aiming to indirectly quantify gRNA activity (33,39); they define a phenotypic outcome. Such experimental methods are relatively easy to perform, but they usually underestimate the actual CRISPR gRNA activity and could produce artifacts in the training data. For instance, equally efficient Cas9 cleavage sites may not result in comparable phenotypic changes, as demonstrated in (77). The authors of (77) observed that the performance of the predictive models trained with flow cytometry data was better than those trained with resistance assay data, indicating that there is substantial heterogeneity among different experimental measurements.

However, guide activity can also be captured directly using deep sequencing (78). This method measures exactly the presence of mutations introduced by CRISPR–Cas9 at the relevant target site. Despite its accuracy, this is a costlier approach and does not provide information about phenotypic outcome. Furthermore, the endogenous DNA repair machinery might affect the readout of the sequencing methods.

To further complicate the process of data labeling and model training, gRNA activity is sometimes represented in public datasets using a discrete variable (high/low) and in others using a continuous one (typically taking values in the range of 0–100%). In the latter case, a regression model can be trained to predict the exact gRNA efficiency, while in the discrete case, a classification model can predict whether that gRNA is active or not. The two types of models are suitable for different use cases and are hardly comparable. Although classification is potentially less informative than regression, exact prediction of gRNA efficiency is a much harder problem. This fact is supported by our analysis that shows actual CRISPR efficiency to be only weakly correlated with classifiers’ predictions. Limited sample sizes and incomplete feature sets favour coarse-grained high/low classifications which are more accurate, albeit less informative than continuous efficiency predictions. Therefore, the ability of classification algorithms to differentiate between highly active and less active gRNAs remains valuable as a stopgap solution until regression-based tools can more accurately model gRNA efficiency.

#### Features for optimal gRNA efficiency

Designing and selecting meaningful gRNA features for efficiency prediction is a difficult process. This process also requires good knowledge of the CRISPR gene editing mechanism which has not been fully resolved yet. Moreover, feature identification can extend to hypothesis generation; extracting and ranking relevant features can promote the design of CRISPR experiments to further elucidate the gene editing mechanism. Deep learning can facilitate the feature identification procedure, through representation learning. In this section, we compare the results of automated feature identification through deep learning to the features discussed in the Introduction (Table 1). Table 7 presents the features that were identified in three previous studies using deep learning models (41,53,73).

**Table 7.** Guide RNA features based on Deep Learning models

Categories/models	**Efficient features**	**Inefficient features**
*Overall nucleotide usage*		
DeepCRISPR	A, U in the middle	C in the middle
CNN-SVR	A, U in the middle	G count
C-RNNCrispr	NN in positions 17–19	
*Position-specific nucleotides*		
DeepCRISPR	G in position 20	**A in position 20**
	C in positions 17–20	U in positions 17–20
		G in position 16
CNN-SVR	A in position 20	**G in position 20**
	C in positions 17–20	U in positions 17–20
C-RNNCrispr	A in position 20	A in positions 3–6
	C in positions 17–19	
	**U in position 20**	
*Motifs*		
DeepCRISPR	C in PAM (CGG)	T in PAM (TGG)
	G in PAM (GGG)	A in PAM (AGG)
CNN-SVR	**T in PAM (TGG)**	**C in PAM (CGG)**
	**A in PAM (AGG)**	**G in PAM (GGG)**
*Epigenetic*		
DeepCRISPR, CNN-SVR,	Open chromatin (esp. position 17)	Methylated DNA [RRBS]
C-RNNCrispr	[CTCF, DNase]	

CTCF: CCCTC-binding factor, RRBS: reduced representation bisulfite sequencing, N: any nucleotide.

Conflicting results are marked in bold.

We observe that different - and sometimes contradicting - features are selected by deep learning models. For instance, uracils (U) were found to be disfavored at the four positions closest to the PAM by *DeepCRISPR* and *CNN-SVR*, which is consistent with the fact that multiple Us in the spacer lead to low sgRNA expression (33). However, *C-RNNCrispr* favored the presence of U at position 20. Similarly, *DeepCRISPR* favored cytosine (C) and disfavored thymine (T) in the variable nucleotide of the PAM, which is consistent with the results of previous studies. In contrast, *CNN-SVR* produced the opposite result; T was favored and C was disfavored.

Having said that, there are certain features that were recognized by all three models and they coincide with previous findings. In particular, position 17 has a consistent preference for C, which is the DNA cleavage site of the CRISPR system. There is also a general preference for open chromatin structure, as indicated by CTCF and DNase, and a relative avoidance of DNA methylation, as shown by the RRBS assay. Finally, we note that most of the top features were generated from the seed region of the gRNAs. This observation coincides with previous finding that a prototypical 10–12 nt PAM-proximal seed sequence largely determines target efficacy. We conclude that, although deep learning may help in extracting features, its results need to be functionally validated, in order to confirm their importance for CRISPR activity prediction.

#### Algorithm selection

Previous studies have shown that proper algorithm selection is crucial in training robust models to predict gRNA efficiency (39). According to the literature, linear regression has achieved some success (38), but the more successful models use more complex approaches such as Random Forest (59), Support Vector Machines (36,37), and Gradient Boosted Regression Trees (39), which consider interactions between the individual features (79).

On the other hand, the strength of deep learning is the automated identification of important features through representation learning. Such approaches have undoubtedly improved the state-of-the-art in speech recognition, visual object recognition, object detection, and many other domains (62). However, the search space for CRISPR targets is much smaller than the typical image analysis task. An image may consist of millions of pixels, with objects at different scales, locations, and orientations, while a typical CRISPR target consists of 20–30 bases, with known coordinates for objects such as the gRNA target and PAM. This reduces the potential benefits of deep learning approaches over more traditional machine learning ones. In addition, current public datasets have only tens of thousands of guide sequences, which are often insufficient for training a deep learning model. Nevertheless, this situation may change as different Cas enzymes and more complex applications are being considered, including genome-wide search for optimal targets.

Hence, we conclude that deep learning is but one tool in the toolbox and the right machine learning approach may differ per task. This was demonstrated by the performance of the simple *Azimuth 2.0* algorithm in our analysis, and has also been argued in the literature, for example, in the case of *CRISPR-GNL* (80), a Bayesian ridge regression solution that outperforms its deep learning counterpart, *DeepCas9*. Therefore, choosing the proper algorithm depends on the task and may not be based solely on predictive accuracy. In particular, interpretability, the ability to identify important features, and their interactions are all key factors that can influence the final choice. In the case where more than one algorithm is applicable to a problem, comparisons and benchmarks are often appropriate to identify the optimal solution.

### Highlights

Our analysis shows that the predictive ability of each model varies considerably for different test sets, especially when using zebrafish test data. This confirms previous findings that the optimal on-target efficiency prediction model strongly depends on whether the guide RNA is expressed from a U6 promoter or transcribed in vitro with the T7 promoter (8). No single model outperformed all others across datasets, suggesting that a careful selection of CRISPR gRNA design tools per task is necessary.

The efficiency of a guide RNA is a complex interplay of factors such as sequence composition, secondary structure, and numerous others that are yet unknown. This fact strongly influences the performance of different models and determines the type of data and the task for which it can be used. In general, we show that learning-based tools can tailor the model to particular data, despite the limited biological knowledge of the CRISPR–Cas9 mechanism. More importantly, they can extract additional characteristics and make plausible hypotheses for further investigation. For those reasons, learning-based tools generally perform better than hypothesis-driven ones and should be preferred.

Interestingly, the selection of machine learning algorithm is not the most important design choice. We observed that simpler machine learning tools can outperform some of the new deep learning ones. Large datasets, obtained with uniform, unbiased experimental measurement, play a crucial role in training a generalizable model, as in the case of *DeepSpCas9* and *DeepHF*. Additionally, data labels based on sequencing, rather than phenotypic outcome, may lead to more accurate models, albeit at a cost (59). Therefore, more time should be spent collecting and correctly labeling datasets, designing and extracting meaningful features.

It also follows from our study that the predictive ability of each tool will vary depending on its intended use. If the model is applied to data that are similar to its training dataset, it will likely achieve an acceptable performance. That is not always the case, though, making systematic evaluations valuable and necessary. Because the training dataset has such a strong influence on the final model, it is critical to know the characteristics of the data a model was trained on. As a rule of thumb, models trained on phenotypic data are better suited to identifying target sites that induce functional changes but are limited to tasks relating to the same condition as the training set. In contrast, models trained on sequencing data are more universally applicable, but are only capable of predicting genotype changes, rather than their functional result. It is therefore important to select carefully the appropriate model for a particular task (test dataset), based on the attributes of the experimental design. If such a model is not available, a generalizable tool (e.g. *DeepHF*) or a meta-tool (e.g. the average in our study) could be used to obtain a decent predictive performance.

It is critical for users of gRNA efficiency prediction tools to know which one best suits their research. It is also critical to use the appropriate metric when evaluating the available models to make that decision. Without a good evaluation criterion, it is not possible to choose the best model or features. In this study, we evaluated current tools using two different metrics and obtained very different results. Thus, users should select carefully the appropriate evaluation metric before moving to model selection. The choice of metric depends mainly on the intended use of the model’s predictions, such as large scale knockout screens or targeted gene therapy.

Finally, extracting meaningful, reproducible design rules is not trivial and is further complicated by data heterogeneity. Specifically, we show that studies lead to different and sometimes even conflicting results about the importance of predictive features. Existing public datasets about gRNA efficiency may suffer from a number of biases, creating confusion and preventing unimpaired transfer of the underlying features to other datasets.

To derive reproducible design rules, effective integration of data from different cell types and assays is required. However, it is not clear how that process should be done nor how much it will improve the trained models. An alternative solution would be to carry out large-scale CRISPR activity experiments under standardized experimental conditions, instead of merging CRISPR activity datasets measured by different methods. This will reduce data heterogeneity at the cost of time and resources. On the other hand, it is worth noting the confirmation of several reliable features, such as the GC content, the seed region, and the secondary structure of gRNAs, which can be used as a starting point for good design rules.

In summary, there is little consensus between existing tools for gRNA efficiency prediction. We have identified a number of challenging design issues, which need to be addressed. Adequately addressing these issues will likely translate to improvements in guide design, paving the way for more precise and efficient genome editing using the CRISPR–Cas9 system.

### Conclusion

The CRISPR–Cas9 technology has rapidly emerged as a state-of-the-art technology for functional genome-editing studies. Because of its simplicity, efficacy, specificity, and versatility, this technology has tremendous advantages over other gene-editing technologies. The machine learning gRNA design tools serve as an important platform for the efficient application and development of the CRISPR system. However, the existing models still have some flaws, such as their sensitivity to data heterogeneity, unclear mechanism of decision making, insufficient training datasets and inadequate ability to produce general gRNA design rules. Hence, further efforts are required to improve *in silico* gRNA design with high on-target activity and reduced off-target effects.

## MATERIALS AND METHODS

### Data collection and processing

This section describes the process we followed to collect, extract and prepare the data used in our study.

#### Training datasets

We used ten public experimentally-validated gRNA efficiency datasets, which were collected and processed by Haeussler *et al.* (8). These datasets cover several cell types of five organisms, some of which have been used to develop existing tools and algorithms. We kept only five of those to train and develop our baseline model, based on the performance of each XGB model under 10-fold cross validation. We also excluded all the zebrafish datasets to create a model with consistent gRNA design rules. The selected datasets were:

- The Chari dataset, consisting of 1234 guides targeting HEK293T cells with scrambled SpCas9 targets, whose target mutation rates were the readout for efficiency (37).
- The Wang/Xu dataset, consisting of 2076 guides targeting 221 genes whose deletion resulted in a growth disadvantage in the leukemia cell line HL60. The knockout efficiency of this dataset was evaluated based on the decline in abundance in the screens (34,35).
- From the Doench dataset, only 951 guides targeting six cell-surface proteins genes in mouse-EL4 cells, which could be assayed by flow-cytometry, were kept. There the abundance of integrated gRNAs in FACS-isolated, target-negative cells was used as a measure of knockout success (33).
- A new version of Doench *et al.* dataset, consisting of 2333 guides targeting eight human genes in A375 cells whose knockout success was inferred from resistance to one of three drugs (vemurafenib, 6-thioguanine and selumetinib) (39).
- The dataset from Hart *et al.*, consisting of 4239 guides, targeting 829 genes determined to be essential in human HCT116 cells (8,81).

In addition, we extracted two zebrafish datasets (i.e. ‘Shkumatava’ and ‘Gagnon’) to use in our evaluation as independent data (8). To select only the required sequences for each dataset, the ‘dataset’ column was used, which contained the study name. Using these names, seven files corresponding to the seven datasets mentioned above were created.

However, each dataset had its own measurement scale for knockout efficiencies, producing datasets with non-standardized efficiency measurements. Thus, it was necessary to rescale each of these values to a standard measurement scale. To accomplish this, we applied a Min–Max rescale procedure to the cleavage efficiency of each dataset. The Min-Max normalization maps a value in the range [0, 1] via the following equation:

$$\begin{eqnarray*} Y_{norm} = \frac{Y - Y_{min}}{Y_{max} - Y_{min}}, \end{eqnarray*}$$

where *Y*_*min*_ and *Y*_*max*_ are, respectively, the minimum and maximum efficiency value of the dataset, *Y* is the original efficiency and *Y*_*norm*_ is the normalized value. This rescaling function was applied on each efficiency measurement of the five training datasets.

Moreover, some datasets included sequences that were shorter than 23-nt, meaning that these sequences were not in the form of a 20-nt sequence followed by a 3-nt PAM, leading to non-standardized sequences. For example, the Doench A375 and Hart datasets comprised sequences of length 20-nt. Therefore, we extracted 23-nt sequences in the appropriate form from the provided sequence context. Specifically, the 20-nt sequence was found in the long 100-nt sequence and then extracted along with the 3 following nucleotides, obtaining a 23-nt sequence. Subsequently, we extracted the 30-nt sequences in a similar fashion. Finally, we used the provided *DeepSpCas9* training dataset (71), without any changes.

#### Testing datasets

A total of six testing datasets were gathered from published studies, namely: Labuhn (40,41), Shalem (33), Koike-Yusa (8), Xi Xiang (74), Shkumatava (8) and Gagnon (8). We chose these datasets as they were not used to train the models we evaluated. Thus, they allowed for a fair and unbiased comparison.

The Labuhn dataset was provided in the supplementary material of Chuai *et al.* (41) and was used without further changes. This dataset was reported recently, utilizing fluorescent reporter knockout assays with verification at selected endogenous loci for gRNA knockout efficiency measurement. The dataset contains a total of 424 gRNAs for HEL cells (40).

The Shalem dataset was provided by Doench *et al.* (33) and contains 1278 gRNAs targeting 414 genes. The gRNA efficiency is expressed as the log_2_ fold change in abundance during two weeks of growth in A375 cells.

The Koike-Yusa dataset was provided by Xu *et al.* (35). It originally contained 87,897 gRNAs targeting 19 150 mouse protein-coding genes in mouse embryonic stem cells (mESCs) (82). By posterior analysis, 311 essential genes were identified and 1064 gRNAs were retained (35). We extracted 23-nt and 30-nt sequences from the Shalem and Koike-Yusa datasets using our custom scripts (provided in Supplementary material). We also calculated the mean log_2_ fold change value from Koike-Yusa and used this value for the correlation analysis of gRNA predictions.

The dataset from Xiang (74) was preprocessed by removing gRNAs supported by <200 reads and by intersecting the datasets of gRNAs with efficiencies measured at day 8 and day 10, thus retaining data for 10 592 gRNAs. The mean of the efficiencies measured at day 8 and day 10 was used for the comparative analysis.

The Shkumatava and Gagnon datasets were obtained from Haeussler *et al.* (8). They include a set of 163 and 111 guide sequences in zebrafish, respectively. Guides were transcribed *in vitro* with the T7 RNA polymerase kit and their efficiency represented the number of mutated sequencing clones obtained from zebrafish embryos. After extraction, we found two identical sequences with different efficiencies in the Shkumatava dataset; we kept only one and used their average as its final efficiency.

#### Datasets for epigenetic features

The four human datasets that we used to study the epigenetic features were integrated and processed by Chuai *et al.* (41) and provided by Zhang *et al.* (53). These datasets were originally collected from public datasets (34,39,81). They covered gRNAs targeting 1,071 genes from four different cell lines, including HCT116 (4239 samples) (81), HEK293T (2333 samples) (39), HELA (8101 samples) (81) and HL60 (2076 samples) (34). Each entry in the datasets contained the 23-nt gRNA sequence, four kinds of corresponding symbolic epigenetic features, as well as numerical and binary cleavage efficiency. The gRNA efficiency measurements were restricted to experimentally-validated assays, where the efficiency was defined as the log-fold change in the measured knockout efficiency. The epigenetic features included CTCF and H3K4me3 information obtained from the ChIP-Seq assay, chromatin accessibility information from the DNase-Seq assay, and DNA methylation information from the RRBS assay.

We used these features (sequence, epigenetic, numerical efficiency) to search for identical gRNAs among all pairs of cell lines and extract their epigenetic information. Our processed datasets, thus, include all pairs of identical sequences with their corresponding epigenetic features and can be used to confirm our analysis (Supplementary Tables S1–S3).

### Activity prediction tools

*DeepCRISPR* (41) predictions were generated using the command-line version of the tool, with sequence features only, since the epigenetic features of the tested cell lines were not available in ENCODE (83). Hence, all the tools were compared on the same task using sequence-only information. The source R code of *DeepCas9* was downloaded from https://github.com/lje00006/DeepCas9 and used with the provided weights (69). *DeepSpCas9* prediction results were computed using the authors’ web-based implementation available at http://deepcrispr.info/DeepSpCas9. *DeepHF* (72) predictions were generated using the source code available at https://github.com/izhangcd/DeepHF. Regarding *CRISPRLearner* (https://github.com/pierclgr/CRISPRLearner), a different model for each dataset was used. Specifically, we used the trained ‘Doench mEL4’ and ‘Chari 293T’ model for the Koike-Yusa and Xi Xiang predictions, respectively. In addition, the ‘Moreno-Mateos Zebrafish’ model was used for the Shkumatava and Gagnon datasets. Due to reproducibility issues, we retrained the ‘Wang-Xu HL60’ and ‘Doench Hg19’ models and used them to obtain predictions for the Labuhn and Shalem dataset, respectively. We also retrained *CNN-SVR* and *C-RNNCrispr* using only sequence-level features following the training process the authors described in their studies. *Azimuth 2.0* prediction results were retrieved using the source code available at https://github.com/microsoftResearch//azimuth (39). For a fair comparison with the other algorithms, we did not specify the optional parameters which determine the position of the guide within the gene. *E-CRISP* (Version 5.4) predictions were obtained using their web platform. We applied the provided evaluation feature of the tool on our datasets with default options (‘number of 5′ mismatch positions ignored by the program’ and ‘tolerated edit distance to the target sequence’ were set to zero), after selecting the appropriate organism. We processed the results in a suitable format and integrated all the predictions into a single file for further analysis. All the tool implementations were tested for functionality in January 2022. We also provide example prediction scripts for each tool at https://github.com/VKonstantakos/CRISPR-Deep-Learning.

### Baseline model implementation

We trained 6 Extreme Gradient Boost (XGB) models on 6 different datasets. The datasets consisted of the five datasets extracted from Haeussler *et al.* (8) and the one that DeepSpCas9 used (71). We did not create any manual features but instead represented the sequences using One-Hot-Encoding. Hyperparameters were also not optimized; we used the default XGBRegressor with objective function ‘reg:squarederror’. We then used the six trained models to make predictions on each of the six independent datasets and took their average as the final predicted score. The second baseline model was the average of the predictions we got from *DeepCRISPR*, *DeepCas9*, *DeepSpCas9* and *Azimuth 2.0*. We chose these models because each one implemented a unique training technique and was trained on different datasets.

### Evaluation metrics

Spearman correlation and normalized discounted cumulative gain (nDCG) were used to measure the consistency between experimentally determined gRNA efficiencies and predicted scores. The Spearman rank correlation between two variables is equal to the Pearson correlation between the rank values of those variables. In addition, the Spearman correlation coefficient between two datasets {*x*_*i*_} and {*y*_*i*_} can be computed as:

$$\begin{eqnarray*} \rho _s = 1- {\frac{6 \sum d_i^2}{n(n^2 - 1)}} , \end{eqnarray*}$$

where *d*_*i*_ is the pairwise distance of the ranks of the variables *x*_*i*_ and *y*_*i*_ and *n* is the number of samples. We used Spearman correlation because it does not carry any assumptions about the distribution of the data and is more robust to outliers compared to Pearson correlation coefficient (84). It was also adopted in previous gRNA activity prediction studies (8,39,41,69–71).

In addition, we borrowed nDCG from the information retrieval (IR) literature, in order to capture the ability of each tool to rank sequences correctly, according to their efficiency, without necessarily requesting an accurate prediction of the efficiency itself (85). Similarly, we implemented Precision (86) at different thresholds for the same purpose, which did not prove useful due to the discretization of the continuous actual efficiency into distinct intervals. Besides, Precision does not evaluate the ranking of the relevant documents, in contrast to nDCG. Thus, it was not used for our analysis.

One interesting characteristic of nDCG is that the top results get more attention than the last ones through a discount function. This function can be set to zero for a specific cut-off *k*, whereby the remaining results after the *k*th one are completely ignored (85). This is interesting because we do not want to base our judgment on how well a tool is doing in predicting inefficiency. In our experiments, we set *k* to be the top 20 predicted gRNAs of each dataset. This choice was based on the number of guide RNAs that are typically evaluated for a specific gene. The nDCG value for each tool on each dataset was then calculated as follows: First we obtained a predicted gRNA efficiency rank list, based on the tested tool, where variable *i* represents the *i*-th gRNA in the rank list. Secondly, a variable *rel*_*i*_ was used to represent the relevance of the *i*th gRNA, where *rel*_*i*_ was the real efficiency score of the corresponding gRNA on the test dataset. Finally, the performance of the tool, when considering the top *k* gRNAs, was measured by *nDCG*_*k*_ using the following formula:

$$\begin{eqnarray*} nDCG_k = \frac{DCG_k}{IDCG_k}, \ \textrm {where} \ DCG_k = \sum _{i=1}^{k} \frac{rel_i}{log_2(i+1)} \end{eqnarray*}$$

is the discounted cumulative gain (*DCG*_*k*_) for the obtained rank list and *IDCG*_*k*_ is the ideal discounted cumulative gain (i.e. a perfect ranking algorithm has *nDCG*_*k*_ = 1.0). Spearman correlation was calculated using the Scipy library, while nDCG was calculated with the provided Python script for *k* = 20 using the equation above.

## DATA AVAILABILITY

The final datasets including each model’s predictions are available in Supplementary material (Supplementary Tables S4–S9). In addition, all the used datasets together with the saved models and Python scripts to reproduce our results are available at https://github.com/VKonstantakos/CRISPR-Deep-Learning.

## Supplementary Material

gkac192_Supplemental_Files

## Figures and Tables

**Figure 9.**
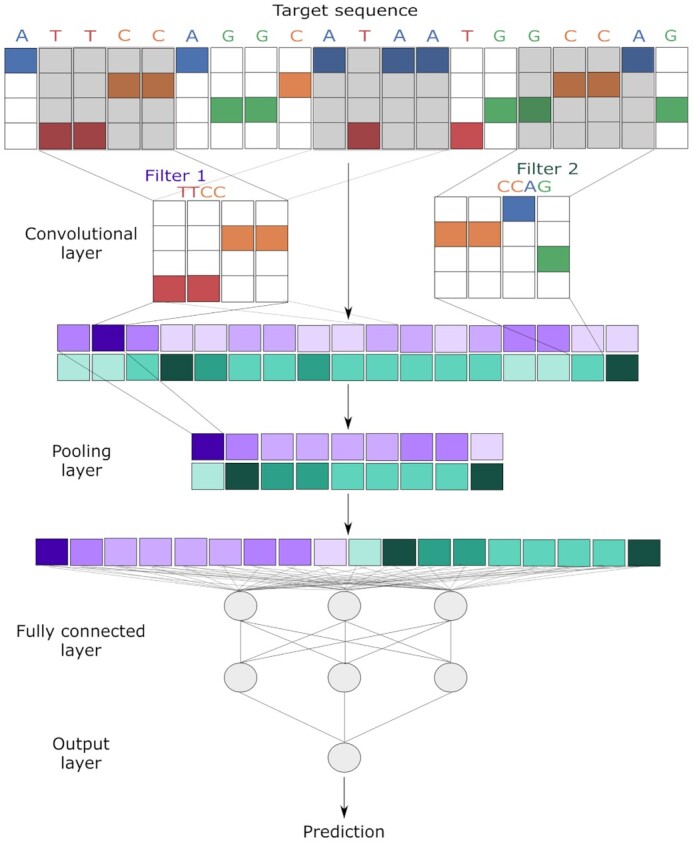
An example of a CNN being used to extract features from DNA sequences. First, the sequence is encoded by ‘one-hot’ vectors with a 1 at the position corresponding to the nucleotide type (A, C, G or T), and zero otherwise. The convolution and pooling operations are then applied to the input vectors to produce the output of each layer as a feature map. In this example, the convolutional layer includes two (4 × 4) filters that detect sequence motifs, while the pooling layer aggregates their output using the maximum pattern match. Finally, the output of the fully connected layers is fed to a regression layer that assigns a score to the given sequence.

## APPENDIX A. CONVOLUTIONAL NEURAL NETWORKS

Convolutional neural networks (CNNs) (62), a class of ANNs, have lately gained popularity in analyzing image-based data. They were inspired by Hubel and Wiesel’s seminal work on the cat’s visual cortex (87), which showed that simple neurons respond to small motifs in the visual field and complex neurons respond to larger ones.

The basic CNN architecture includes three types of layers: convolutional layers, pooling layers, and fully connected layers (Figure 9) (62). Convolutional layers aim to learn feature representations of the inputs. Similar to the cortical neurons, they contain a set of learnable filters that have a small receptive field but extend through the full depth of the input volume. The receptive fields of different neurons (filters) partially overlap such that they cover the entire visual field (input). These filters scan the provided input for local patterns and pass the results to the next layer. Different filters might, for example, detect edges in an image, or sequence motifs in a genomic sequence.

The pooling layers of the CNN, on the other hand, determine the presence of a pattern in a region by calculating the maximum or average pattern match, thereby aggregating information from the whole region into a single number. This way, pooling layers merge semantically similar features into one, reduce the dimensions of the representation, and create an invariance to small shifts and distortions.

A CNN typically consists of several convolutional and pooling layers, allowing to learn more and more abstract features at increasing scales from small edges, to object parts, and finally entire objects. After the successive application of convolutional and pooling layers, there may be one or more fully connected layers that aim to perform high-level reasoning. They connect all neurons of the last pooling layer to every single neuron of the new layer to generate global semantic information (88). An example of a CNN architecture is shown in Figure 9.
